# Fish and the Thyroid: A Janus Bifrons Relationship Caused by Pollutants and the Omega-3 Polyunsaturated Fatty Acids

**DOI:** 10.3389/fendo.2022.891233

**Published:** 2022-05-27

**Authors:** Salvatore Benvenga, Fausto Famà, Laura Giovanna Perdichizzi, Alessandro Antonelli, Gabriela Brenta, Francesco Vermiglio, Mariacarla Moleti

**Affiliations:** ^1^Department of Clinical and Experimental Medicine, University of Messina, Messina, Italy; ^2^Department of Human Pathology in Adulthood and Childhood “G. Barresi”, University of Messina, Messina, Italy; ^3^Department of Surgical, Medical, Molecular and Critical Area Pathology, University of Pisa, Pisa, Italy; ^4^Division of Endocrinology, Dr. Cesar Milstein Hospital, Buenos Aires, Argentina

**Keywords:** fish, nutraceuticals, omega-3 polyunsaturated fatty acids, eicosapentaenoic acid (EPA), docosahexaenoic acid (DHA), autoimmune thyroid diseases, endocrine disruptors, mercury

## Abstract

Benefits of the omega-3 polyunsaturated fatty acids (PUFA) on a number of clinical disorders, including autoimmune diseases, are widely reported in the literature. One major dietary source of PUFA are fish, particularly the small oily fish, like anchovy, sardine, mackerel and others. Unfortunately, fish (particularly the large, top-predator fish like swordfish) are also a source of pollutants, including the heavy metals. One relevant heavy metal is mercury, a known environmental trigger of autoimmunity that is measurable inside the thyroid. There are a number of interactions between the omega-3 PUFA and thyroid hormones, even at the level of the thyroid hormone transport proteins. Concerning the mechanisms behind the protection from/amelioration of autoimmune diseases, including thyroiditis, that are caused by the omega-3 PUFA, one can be the decreased production of chemokines, a decrease that was reported in the literature for other nutraceuticals. Recent studies point also to the involvement of resolvins. The intracellular increase in resolvins is associated with the tissue protection from inflammation that was observed in experimental animals after coadministration of omega-3 PUFA and thyroid hormone. After having presented data on fish consumption at the beginning, we conclude our review by presenting data on the market of the dietary supplements/nutraceuticals. The global omega-3 products market was valued at USD 2.10 billion in 2020, and was projected to go up at a compound annual growth rate of 7.8% from 2020 to 2028. Among supplements, fish oils, which are derived mainly from anchovies, are considered the best and generally safest source of omega-3. Taking into account (i) the anti-autoimmunity and anti-cancer properties of the omega-3 PUFA, (ii) the increasing incidence of both autoimmune thyroiditis and thyroid cancer worldwide, (iii) the predisposing role for thyroid cancer exerted by autoimmune thyroiditis, and (iv) the risk for developing metabolic and cardiovascular disorders conferred by both elevated/trendwise elevated serum TSH levels and thyroid autoimmunity, then there is enough rationale for the omega-3 PUFA as measures to contrast the appearance and/or duration of Hashimoto’s thyroiditis as well as to correct the slightly elevated serum TSH levels of subclinical hypothyroidism.

## Introduction

After having reminded the benefits of the omega-3 polyunsaturated fatty acids (PUFA) on several autoimmune diseases, we aim to review their benefits in the thyroid setting both at experimental and clinical levels. We start our work by providing a snapshot of fish consumption since fishes represent a major dietary source of omega-3 PUFA. The other side of the coin (or the other face of Janus, upon comparing fish to this god of the Ancient Roman mythology) is that eating fish means also “eating pollutants”. One relevant pollutant is mercury (Hg), a trigger of autoimmunity that is measurable inside the human thyroid. However, in fish the two faces have unequal size. Indeed, in small oily fish, such as anchovy and sardine, the good face represented by the omega-3 PUFA is much larger than the bad face represented by the pollutants. Just the opposite is true for the large, top predator fish, such as swordfish and tuna. Thus, we also provide a review of studies showing thyroid consequences from eating fish. Finally, after having reminded the interactions between the omega-3 PUFA and thyroid hormones at several levels, and having reminded that other natural compounds have shown benefits in the setting of thyroid autoimmunity, we talk about the commercial side of the benefits of all such substances, namely the expanding market of nutraceuticals. Overall, we think that the use of supplements containing omega-3 in the clinical thyroid setting has enough scientific rationale.

## Consumption of fish

The European Market Observatory for Fisheries and Aquaculture Products (EUMOFA) considers fish and seafood as “*the aggregation of finfish, crustaceans and mollusks and cephalopods*”, regardless of being fresh or chilled and frozen (1). Examples of crustaceans are lobsters, shrimps and crabs; examples of mollusks are oysters and clams; examples of cephalopods are octopus, squid, and cuttlefish (1). Household expenditure on fishery and aquaculture products grew by 17% from 2019 to 2020, much greater than the 2.1% inflation of prices for such products (1). This increase was most likely due to the increased at-home consumption due to the closing of the hotels and restaurants because of the COVID-19 pandemic (1). In 2017, the expenditure on fish in the European Union (EU) was equal to euro 54262 million, which was the highest in the world. However, in terms of per capita expenditure, with euro 106 EU ranked 8th after Iceland, Japan, Korea, Norway, Australia, Israel, and Switzerland (1). Excluding out-of-home consumption, household nominal expenditure on fishery and aquaculture products in 2020 (with % variation over 2019 given in parentheses) was led by Spain (euro 13608 million, +39%) and Italy (euro 12277 million, +3%), with Slovenia (euro 90 million, +1%) and Malta (euro 58 million, +4%) at the bottom of the ranking.

Consolidated data for consumption and other items are available up to 2019 (1). Consumption of fishery and aquaculture products in the EU dropped to 12.30 million tonnes of live weight equivalent (LWE) in 2019, continuing a declining trend that started in 2017. Wild products accounted for 9.41 million tonnes LWE (76% of 12.30), and farm products for 2.89 million tonnes LWE (the remaining 24%). Per capita apparent consumption, estimated at 23.97 kg LWE of mostly wild-caught products, was almost stable in 2019 compared with 2018. Portugal remains the major EU consumer, with 59.91 Kg per capita (-2% vs 2018). Portugal is followed by Spain (46.02 Kg, unchanged), Denmark (42.56 Kg, +6%), France (33.26 Kg, -0.5%), Luxembourg (32.84 Kg, -3%), and Italy (31.21 Kg, +1%). At the other extreme, Hungary and the Czech Republic consume 6.28 and 6.0 Kg (+3% and +7% vs 2018, respectively) (1).

Concerning the most consumed fish, 16 products were considered, and they were listed in decreasing order of consumption: tuna, salmon, cod, Alaska pollock, shrimp, mussel, hake, herring, squid, surimi, sardine mackerel, trout, sprat (brisling), saithe (coalfish) and other products. Illustrative per capita consumption in 2019 (with percentage change vs 2018 given in parentheses) were 3.10 Kg (+ 2%) for tuna, 2.11 Kg (-1%) for cod, 1.02 Kg (+ 2%) for hake, 0.98 Kg (-17%) for herring, 0.58 Kg (+1%) for sardine, 0.53 Kg (-12%) for mackerel, and 6.58 Kg (-2%) for other products (1). In 2020, over 80% of the total volume of fresh fishery and aquaculture products consumed by households in 11 EU countries analyzed was accounted for by Spain, Italy, and France. In decreasing order, the top five fresh fish species consumed by households in 2020 were: hake, salmon, sardine, European seabass, and gilthead seabream in Spain; gilthead seabream, mussels, salmon, European seabass, and anchovy in Italy; salmon, cod, saithe (coalfish), trout and gilthead seabream in France. Data for swordfish, a seafood species that will be mentioned subsequently in our review, were not provided.

Based on a document by CBI (Centre for the Promotion of Imports from developing countries) the swordfish fishery is very important for Southern Europe, especially Spain and Italy, the two European countries with the highest consumption of swordfish (2). With 22,676 tonnes in 2017, Spain is the leading European producer of swordfish, followed by Italy and Portugal. The top importers from non-European countries of frozen swordfish are Portugal, Italy and Spain (6,316, 3,762 and 2,130 tonnes, respectively). Italy’s imports of swordfish have increased by 18% since 2014 with most deliveries coming from Spain. Based on a document by Oceana (3), which is the no-profit largest international ocean conservation organization, Greece, Italy, and Spain are the European countries where swordfish is consumed the most, but numbers were not provided. According to a Spanish paper (4), which in turn reports data published in 1995, the consumption of swordfish by Spanish adults averaged 0.35 g/day, with marked differences between communities, ranging from areas of no swordfish consumption to two areas of highest consumption (1.06 g/day and 1.17 g/day). On an annual basis, the said 0.35, 1.06 and 1.17 g/day correspond to 0.13, 0.39 and 0.40 Kg/year. In Italy, swordfish accounts for 4.9% of the national consumption of fresh fish (5), and in the year 2017 it was the fifth species most consumed (5.7%), following European seabass (17%) (6). Thus, of the 29.80 Kg of fish consumed by Italians in 2017, 1.46 Kg were accounted for by swordfish. A recent Italian survey of 560 consumers (7), led to the identification of 24 seafood species that were commonly purchased. The highest preference was for gilthead bream (*Sparus auratus*), European seabass (*Dicentrarchus labrax*), swordfish (*Xiphias gladius*), and European pilchard (*Sardina pilchardus*), with approximately 13% of consumers who purchased swordfish.

## The Good Face of Janus

There is abundant literature, which is accruing over the years, about the benefits of the omega-3 fatty acids as such or omega-3-rich-foods on some disorders (**Table 1**), including autoimmune diseases (8–18). An example of the clinical studies that show such benefits for illustrative autoimmune diseases is summarized in **Table 2** (19–36), with more details provided for type-1 diabetes (T1D) due to the endocrine nature of this disorder. **Table 1** also illustrates the magnitude of the literature on other topics of this review, such as fish consumption.

**Table 1:** Magnitude of the literature retrievable on PubMed at two indicative time points.

Entries	Number of articles	
	as of May 21, 2015	as of Jan 04, 2022
Omega-3 fatty acids	20,521	32,757 (+ 59.6%)
Protective effects of omega-3 fatty acids	768	4,951 (+ 545%)
Protective effects of DHA	297	2,347 (+ 690%)
Protective effects of fish oil	809	5,527 (+ 583%)
Consumption of omega-3 fatty acids	2,070	3,405 (+ 64.5%)
Consumption of fish	10,481	17,546 (+ 67.4%)
Protective effects of fish consumption	214	1,519 (+ 610%)
Omega-3 fatty acids and autoimmunity	36	291 (+ 708%)
Omega-3 fatty acids and autoimmune diseases	433	640 (+ 47.8%)
Fish consumption and autoimmune diseases	56	105 (+ 87.5%)
Fish consumption and autoimmune thyroiditis	3	8 (+ > 167%)
Fish consumption and thyroiditis	Zero	123 (+ > 100%)

**Table 2:** Example literature on the effects of fish/fish oil consumption or supplements containing omega-3 polyunsaturated fatty acids (PUFA) on representative autoimmune diseases. *

Disease	Effects	Reference
**Rheumatoid arthritis (RA)**	Evidence that higher **consumption** of olive oil, **oil-rich fish**, fruit, and vegetables **protect** from the development of **RA**.	Pattison et al (19)
	**Consuming** high amounts of **omega-3 fatty acids and fish** is one of the modifiable factors that are recommended to **prevent the risk** for the development of **RA**.	Koller-Smith et al. (20)
	Compared with the lowest category of **fish consumption**, the highest category was inversely associated with risk of **RA** (RR: 0.89). Furthermore, a 100 g/day increment in **fish** intake was associated with a 15% **decreased risk** of RA.	Asoudeh et al. (21)
	An **intake** of dietary **long-chain n-3 PUFAs >**0.21 g/day (lowest quintile) was associated with a 35% **decreased risk** of developing **RA** (RR= 0.65) compared with a lower intake. Long-term **intake** consistently above 0.21 g/day was associated with a 52% **decreased risk**. Consistent long-term **consumption** of **fish** ≥1 serving per week compared with<1 serving per week was associated with a 29% **decrease in risk** (RR 0.71).	Di Giuseppe et al. (22)
	**RA** patients **consuming fish** ≥2 times/week had a significantly **lower disease activity score** (**DAS**) compared with patients who ate **fish** never or <1 time/month (difference -0.49). For each additional serving of **fish** per week, **DAS** was significantly **reduced** by 0.18.	Tedeschi et al. (23)
	**RA** patients in the **fish oil group** (10 g/day for 6 months) reported a significantly **decreased** consumption of non-steroidal **anti-inflammatory drugs** at 3 and 6 months, and; their **status** of global arthritic activity **improved** at 3 months. In contrast, control patients reported an increased global arthritic activity at 6 months.	Sköldstam et al. (24)
**Multiple sclerosis (MS)**	**Consuming fish/seafood** at least once a week or at least once a month with regular **fish oil use** was associated with 44% **reduced odds** of **MS** compared with consuming **fish/seafood** less than once a month and no **fish oil supplementation**.	Langer-Gould et al. (25)
	Dietary **intake** of at least 0.5 servings of **fish** per week during adolescence and after **could reduce the risk** of **MS**. **Consumption** of **fish decreases the risk** of **MS** [OR= 0.77] compared with controls.	Rezaeizadeh et al. (26)
	Higher total **fish consumption** (30 g/day, equivalent to two servings/week) was associated with an 18% **reduced risk** of central nervous system demyelination (FCD), a precursor to **MS** (adjusted OR 0.82). Higher **tinned fish consumption** (30 g/day) was associated with a 41% **reduced risk** of FCD (adjusted OR 0.59). **Tinned fish** is predominantly **oily**. **Oily fish** is high in vitamin D and **long-chain n-3 PUFAs**.	Black et al. (27)
	Frequent **fatty fish intake** was associated with **decreased occurrence** of **MS** (adjusted OR 0.82). The association between **intake** of **lean fish** and MS was not significant.	Bäärnhielm et al. (28)
**Type 1 diabetes (T1D)**	6081 Finnish newborn infants with HLA-DQB1-conferred **susceptibility to T1D** were followed up to 15 years of age. Over the whole period, the risk of advanced **islet autoimmunity** (IA) in **high fish consumers** tended to be **lower compared to low fish consumers**. The overall hazard ratio of advanced IA for **high fish consumers**, compared to **low fish consumers**, was 0.68 and of **T1D** 0.45. The number of children with **T1D** was 15 (**1.6%,** n= 941) among the **high fish consumers** and 180 (**3.9%,** n= 4604) among the **low fish consumers**, a significant difference (P < 0.001). The potential **benefits of fish consumption** could be related to the **n-3 fatty acids**.	Syrjälä et al. (29)
	75 male Albino rats were divided into three groups of 25 each: a negative control group; a group injected with 150-mg/kg body weight of recrystallized alloxan to induce **hyperglycemia**; a group injected with that dose of alloxan to induce **hyperglycemia** and treated with insulin injection. Each group was divided into five subgroups. The 1^st^ subgroup was fed on a casein diet, while the 2^nd^, 3^rd^, 4^th^ and 5^th^ subgroups were fed a basal **diet containing mackerel, sardines, herring**, and **bolti**, respectively. Feeding diabetic rats with the different types of diet **(fish diet)** resulted in an **improvement of the nutritional parameters** (**serum glucose**, triglycerides, cholesterol, LDL-c, HDL-c, VLDL-c, urea nitrogen, uric acid, transaminases) **decreased** in all treated groups, **especially** in the rats receiving the **mackerel diet** and those receiving **sardine diet**, as compared to the casein diet-fed control rats. Furthermore, **diabetic rats** that were treated with a low insulin dose and fed on the **mackerel diet**, showed **non-significant differences** in any parameter, as compared to **non-diabetic rats**.	Abdel-Megeid et al. (30)
	Review of the molecular mechanisms and signaling pathways induced by **ω-3 PUFAs** and the beneficial effects of **ω-3 PUFAs intake in preventing and treating T1D**. Neonates of **T1D** mothers had lower plasma levels of **DHA** and other fatty acids compared to neonates of non-diabetic mothers. At least two major mechanisms can explain the **benefits** of the **ω-3 PUFA** in **T1D**: **anti-inflammatory** and **anti-autoimmunity action**. One of the several molecular targets for the **anti-inflammatory** effects of **ω-3 PUFA** is **peroxisome proliferator-activated receptor-gamma (PPARγ). PPARγ** activation prevents NF-κB nuclear translocation and **reduces inflammatory responses**. In the **NOD mice** (the murine model of **T1D**), administration of **ω-3 PUFA** resulted in the **modulation** of the differentiation of T helper (Th, CD4+) cells and regulatory T cells (Tregs), also **alleviating the inflammatory burden** by decreasing IFN-γ, IL-6, IL-17, and TNF-α levels. Similar effects were reported on the differentiation of Th cells isolated from human peripheral blood mononuclear cells (PBMC) by reducing the Th1 cells population, **balancing the Th1/Th2 ratio** and **suppressing** IL-17A production, and increasing IL-4 and IL-10 secretion. In the NOD mice, **Daily intake** of **EPA and DHA** (3.6 g/kg b.w., as **fish oil**) for 35 weeks **reduced the incidence of T1D** (**33%** of the treated mice **compared with 80%** of the control group). **EPA** and **DHA decreased** significantly **the incidence of severe insulitis** and **elevated insulin secretion**. In the fat-1 transgenic mouse model, the **islets cells contain higher levels of ω-3 PUFA** and lower levels of ω-6 PUFA compared to non-transgenic cells. The transgenic islets were **resistant to cytokine-induced cell death** when exposed to IL-1β, IFN-γ, and TNF-α.	Purdel C et al. (31)
	**Vitamin D** (1000 IU/day) and **ω-3 PUFA** (**DHA** and **EPA** at 60 mg/kg/day) **co-supplementation** for 12 months improves **T1D** by **attenuating autoimmunity** and counteracting inflammation. The co-supplementation **decreased** significantly **insulin demand**, especially as pre-meal boluses, and insulin-dose adjusted HbA1c.	Cadario F et al. (32)
	The authors explored the **preventative and therapeutic effects of ω-3 PUFA on T1D**. Female **NOD mice** were fed a **diet** enriched in **DHA/EPA** for 35 weeks, starting at 5 weeks of age. Further to a control group fed a regular diet, a separate group of animals was fed a diet containing equal levels of arachidonic acid (AA, a ω-6 PUFA). At a **preventative** level, the **islets** from the **DHA/EPA-fed** mice had a significantly **reduced incidence of severe insulitis** compared with mice maintained on an AA-enriched diet or a regular diet. Furthermore, **ω-3 PUFA modulated the differentiation of Th cells and Tregs and decreased** the levels of IFN-γ, IL-6, IL-17, and TNF-α. To test the **therapeutic effects**, the authors delivered, into NOD mice, a lentiviral vector carrying a modified *Caenorhabditis elegans* cDNA, **mfat-1** (mice that are referred as **lenti-mfat-1**), that encodes an ω-3 fatty acid desaturase. Such **lenti-mfat-1 have elevated endogenous levels of ω-3 PUFAs** with a concomitant decrease in ω-6 PUFAs. Around 3 to 4 weeks after lenti-mfat-1 treatment, **nonfasting blood glucose levels had gradually dropped** in most mice. Simultaneously with the normalization of glycemia, **serum insulin** levels in the **lenti-mfat-1–treated group** and the **DHA/EPA-enriched** diet group were **completely restored**. **Lymphocyte infiltration** of neopancreatic islets in the **lenti-mfat-1–treated** or **DHA/EPA-enriched** dietary group was **much lower** compared with lenti-control-treated groups. A high proportion of **regenerated islets** (~40%) had essentially all β cells, with very few α cells. The authors concluded by underscoring the **clinical potential** of gene therapy or nutritional **supplementation of ω-3 PUFAs -** in particular **DHA** and **EPA** - in **preventing and reversing the development of autoimmunity and T1D**, and **perhaps other autoimmune** diseases.	Bi et al. (33)
	This study, termed DAISY (Diabetes Autoimmunity Study in the Young), involved 1770 children at **increased risk for TID**. The authors investigated a direct association between the **ω-3** or ω-6 **PUFA intake** and the **development of islet autoimmunity (IA)**. They found that the **intake of ω-3 PUFA increased the content of ω-3 PUFA** in erythrocyte membranes. Furthermore, in children with familial **T1D,** the long-term dietary **intake of ω-3 PUFA** starting from 1 year of age was shown to be associated with **a reduced risk of IA**.	Norris et al. (34)
	Male Wistar rats were injected with citrate buffer (control group) or 55 mg/kg streptozotocin (STZ). Control and diabetic groups (STZ) were fed with n-6/n-3 ratio of ≈ 7, STZ + n6 (2.5% sunflower oil) with n-6/n-3 ratio ≈ 60 and STZ + **DHA** with n-6/n-3 ratio of ≈ 1 containing 19% **DHA** and 16% **EPA**. Extensive vacuolization of distal tubular cells (DTCs) was found in **T1D**, but it was **attenuated** in the STZ + **DHA** group, which had the highest renal NF-kB expression. The ectopic lipid accumulation was observed in proximal tubular cells (PTCs) of all **diabetic** animals, but it became worse in the STZ + n6 group. Thus, the early phase of **diabetic nephropathy** is characterized by extensive damage and vacuolization of DTCs, which could be **attenuated by n-3 PUFA supplementation**.	Vitlov Uljević et al. (35)
	Participants with **previously diagnosed T1D supplemented their diet** with a 10 mL dose of **seal oil ω-3 PUFAs** containing 2,330 mg of essential fatty acids (1,020 mg **DHA**, 750 mg **EPA**, 560 mg docosapentaenoic acid [DPA]) for 12 months. Of the 40 participants enrolled, aged 48 ± 14 years, with **T1D** duration of 27 ± 18 years, 32 completed the full 12-month protocol. Baseline **corneal nerve fiber length (CNFL)** was 8.3 ± 2.9 mm/mm^2^ and i**ncreased** significantly to 10.1 ± 3.7 mm/mm^2^ after **supplementation**. **Corneal nerve branch density (**CNBD**)** also **increased significantly** (from 10.6 ± 12.5 to 19.6 ± 19.7 br/mm^2^). Furthermore, 12 months of **seal oil ω-3 PUFA prevented the progression** of clinical disease **symptoms and prevented declines** in small and large sensory fibers and functional measures.	Lewis et al. (36)

*Keywords of relevance are highlighted by the bold-face print.

**Figure 1:**
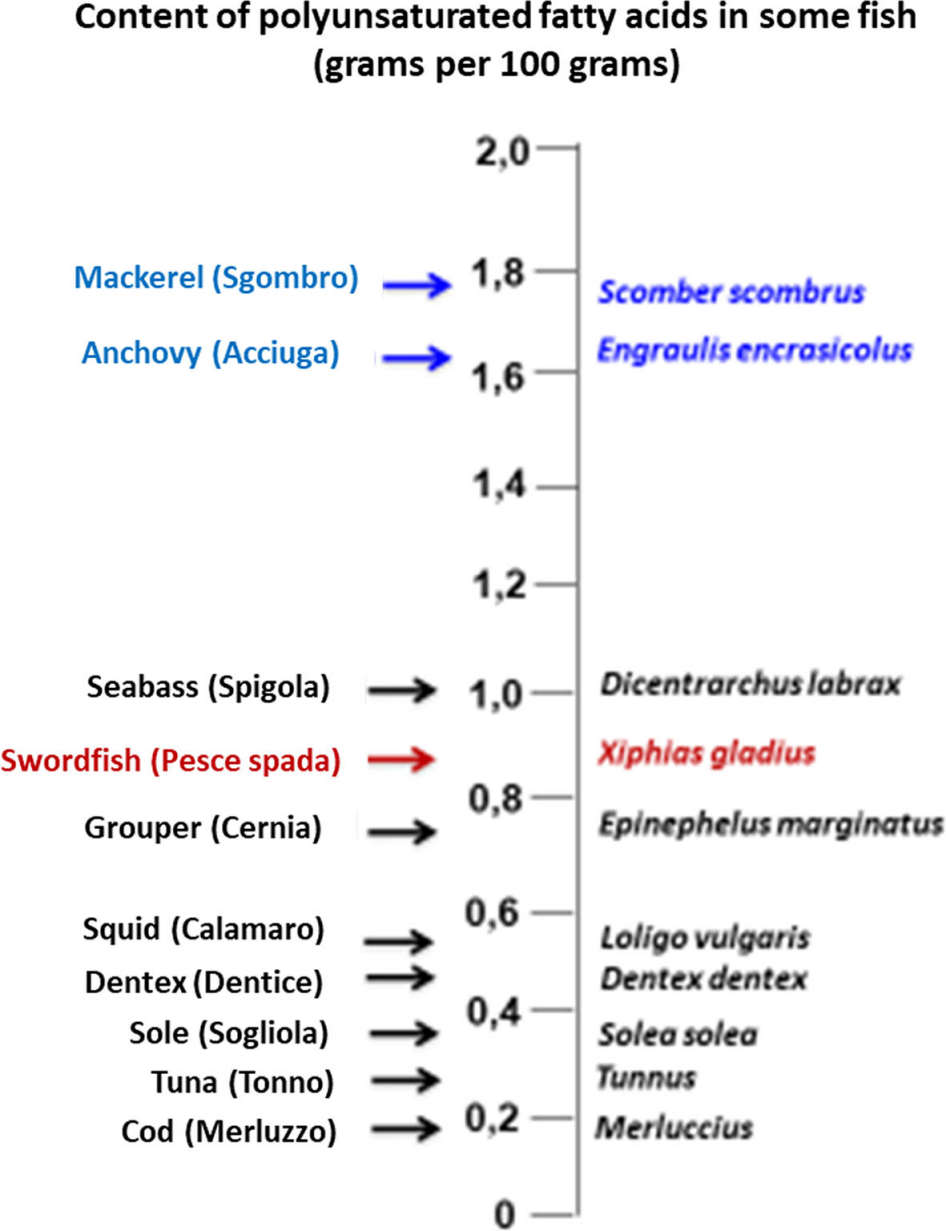
Content of polyunsaturated fatty acids in illustrative species of fish. Oily fish (which are referred to as “*pesce azzurro*” [“azure fish”] in Italy) are typed in blue, while swordfish (the top predator fish that is mentioned more frequently in our review) is typed in red.

**Figure 2:**
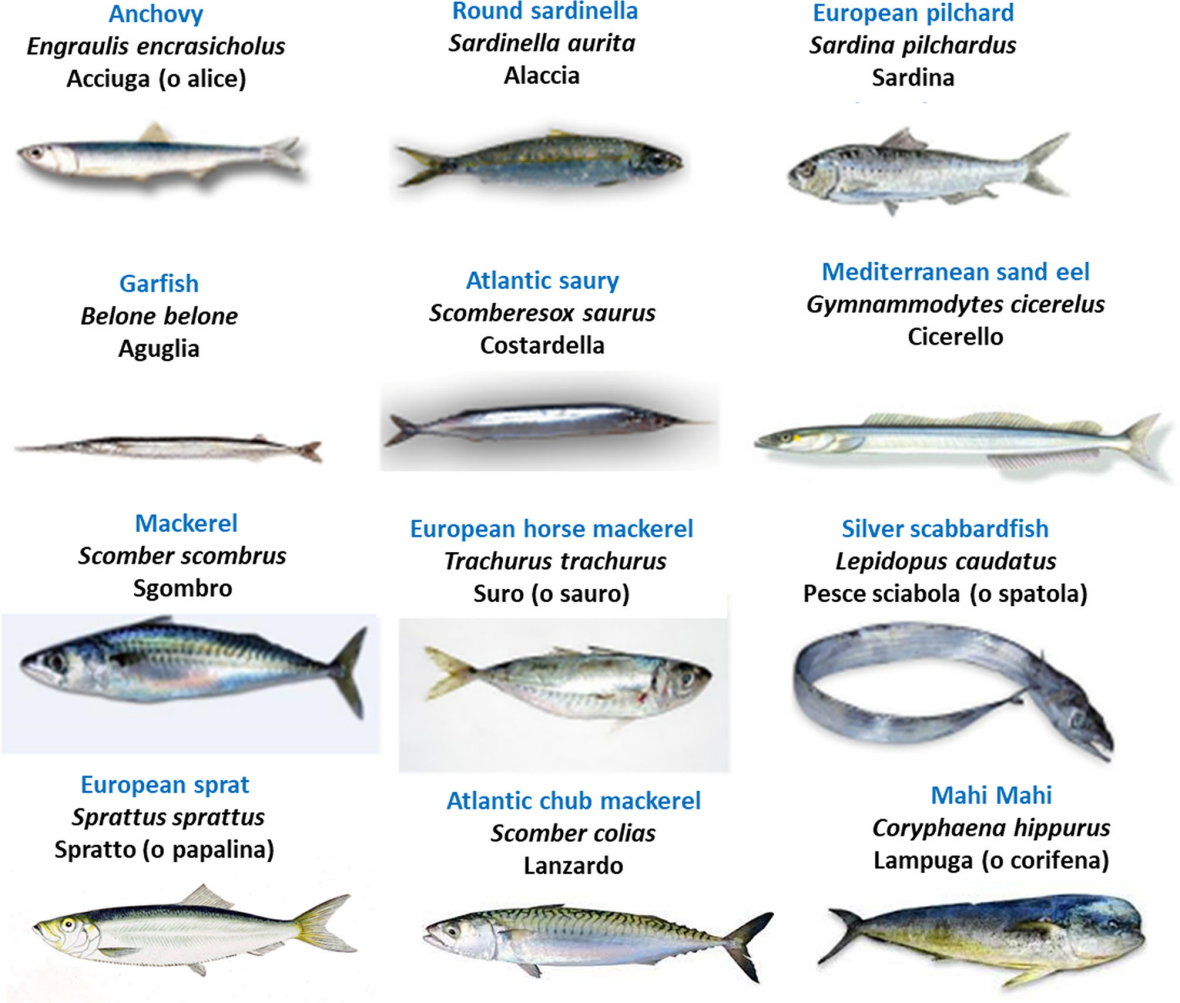
Illustration of oily fish (“*pesce azzurro*” [“azure fish”] in Italy), with the English names typed in blue, Italian in black, and scientific/Latin name in *italics*. Source: https://it.wikipedia.org/wiki/Pesce_azzurro.

As well known, fish (particularly seafood) is a good source of high-quality proteins, vitamins, and minerals, including minerals relevant to thyroid physiology (iodine and selenium). As also well known, fish is a major source of the long-chain omega-3 polyunsaturated fatty acids (abbreviated as either LC n-3 PUFA or omega-3 PUFA), particularly docosahexaenoic acid (DHA) and eicosapentaenoic acid (EPA). **Figure 1** illustrates the content of EPA and DHA in representative seafood (17) while **Figure 2** illustrates the oily fish (with their scientific, English and Italian names), which will be referred to in some sections of this review. Italian names are reported because in Italy we refer to the oily fish as “*pesce azzurro*” (“azure fish”) due to the blue-green color of the green in the dorsal and later scales (37). In the absence of a scientific definition, their definition is culinary (37). In contrast, the n-6 (omega-6 PUFA), such as arachidonic acid and linoleic acid, are derived largely from plant sources. Plants (and therefore plant oils and seeds) are also a source of another LC n-3 PUFA: α-linolenic acid (ALA). Tissues can convert ALA into EPA and DHA, but because the corresponding conversions are only 1 to 10% and 0.5-5% in human tissues, this conversion process is inefficient in humans (38). Thus, the main dietary source of EPA and DHA is cold-water oily fish such as mackerel, salmon, herring, sardines, anchovies, etc…. Canned fish contains omega-3 PUFA, but some amounts may be removed during processing (39). A similar decline in content of the omega-3 PUFA occurs with frying (39, 40).

## The Bad Face of Janus

Fish and seafood contain many contaminants, since anthropic activities (e.g., agriculture, industry, mining) increase their concentration in the aquatic ecosystem (17, 41–43). Clearly, because of biomagnification, pollutants are mostly concentrated in the longer-living and larger predatory fish species such as swordfish, tuna, and shark. Classical fish contaminants are the heavy metals, with Hg being worthy of mention in this review because it acts as an environmental trigger of autoimmunity (17, 44–51), a mechanism that has also been observed for cadmium (Cd) (51–55). In predatory marine fish, approximately 90% of Hg is methylated (methylmercury), while the remainder consists of minimal amounts of inorganic mercury, ethylmercury, and phenylmercury (4).

A recent Italian study on the muscle tissue of 30 Mediterranean swordfish determined that the rank order of toxic metals was Hg, Cd and lead (Pb), while the rank order of essential metals was zinc (Zn), copper (Cu), nickel (Ni) and chrome (Cr) (56). Particularly, Hg, Cd, and Pb levels exceeded the respective critical values 1.02, 0.30 and 0.25 μg/wet weight (that is, the legal safety limits established by the European Community) in eight, three, and two of the swordfish specimens examined, respectively. In a Canadian study, Hg was detected in all samples of swordfish, tuna, marlin, and shark purchased from major supermarket outlets and fish retailers, with swordfish containing the greatest concentration (57). In a Food and Drug Administration document on “*Mercury Levels in Commercial Fish and Shellfish*”, where 68 types of fish are ranked based on their mean content of Hg, the 68th, 67th and 66th positions are occupied by tilefish (1.123 parts per million [ppm]), swordfish (0.995 ppm) and shark (0.979 ppm]) (58). In contrast, sardine and anchovy occupy the 5th and 8th positions with a mere 0.013 and 0.016 ppm concentration of Hg, respectively (58). Thus, it is no surprise that the blood Hg concentrations of 285 adult seafood consumers in Long Island (NY, USA) were positively associated with weekly tuna steak or sushi intake and monthly or weekly swordfish, shark, or marlin intake (59).

With particular reference to thyroid autoimmunity, associations between total blood Hg and positive thyroid autoantibodies (thyroglobulin autoantibodies [TgAb] and thyroperoxidase autoantibodies [TPOAb]) were evaluated using the National Health and Nutrition Examination Survey (NHANES), 2007-2008 (60). Such associations were searched in 2,047 non-pregnant, non-lactating women. Compared to women with the lowest Hg levels (≤0.40 μg/L), those with Hg levels >1.81 μg/L (upper quintile) had 2.24 greater odds (95% CI=1.22, 4.12) for TgAb positivity. In contrast, no significant association was found for TPOAb positivity (60). One study from the Czech Republic in patients with Hg hypersensitivity (61) concluded that removal of Hg-containing dental amalgam may help to successfully treat autoimmune thyroiditis (AIT). In this study, 27/39 AIT patients had Hg hypersensitivity (with amalgam fillings removed only in 15/27), while 12/39 AIT patients had not (controls). Serum TgAb and TPOAb were measured both at baseline and six months later. Compared to baseline, the 15 patients in whom amalgam fillings were removed had a significant decrease in the serum levels of both TgAb and TPOAb (P=0.0007). In contrast, both TgAb and TPOAb did not change in the other two groups (61). Also, for Cd, the other heavy metal that we mentioned above, a direct relationship with thyroid autoantibodies was reported (53). In Chinese women, natural log-transformed blood levels of Cd correlated directly with natural log-transformed serum levels of TgAb (53).

There is evidence for the Hg presence in the thyroid. In a very recent autopsy-based Australian study (62), the presence of intracellular inorganic Hg was searched in paraffin-embedded thyroid tissue blocks from 115 persons (68 males, 47 females; mean age = 54 years, median age = 47 years, range= 1 to 104) with varied clinicopathological conditions. Using autometallography, Hg was found in the thyrocytes with an age-dependent frequency: 4%, 9%, and 38% of persons in the age band 1-29, 30-59, and 60-104 years, respectively. The frequency of thyroid samples containing Hg was similar in males (18%) and females (21%). Laser ablation-inductively coupled plasma-mass spectrometry not only confirmed the presence of Hg, but also detected other metals in six selected samples: cadmium (n= 6), iron (n= 5), lead (n= 4), nickel (n= 2), and silver (n= 2). The authors concluded that Hg can trigger genotoxicity, autoimmunity, oxidative damage, and be involved in the pathogenesis of AIT, hypothyroidism, and thyroid cancer (TC) (62). A previous Italian study on thyroid tissue samples removed at surgery from 77 euthyroid subjects showed that Hg and Cd are significantly more concentrated in the thyroid than in the adjacent muscle and fat of the same individual (63). Worthy of note, this Italian group (64) compared the urine concentration of several metals in a Sicilian area that features an incidence of thyroid-cancer two-fold greater than a control area. The authors found that the geometric mean value in the first area was at least two-fold higher than that in the second area for eight metals, two of which being Hg and Cd (64).

Having mentioned TC is not improper, given the abundant literature on the important predisposing role for such malignancy (particularly, papillary TC) and its advanced stages exerted not only by Hashimoto’s thyroiditis (HT) but also by serum TSH *per se*, even by TSH levels that are within the upper values of the reference limits (65–88). In regard to TC, it is also pertinent to remind the involvement of chemokines (89–98) and the peroxisome proliferator-activated receptors (PPARs) in the molecular oncogenesis (95–106), so that both types of molecules may serve as novel targets of TC precision therapy or prevention. Quite interestingly, the beneficial health effects of DHA and EPA on metabolic diseases are thought to arise from their binding to and activation of PPARs (107), as it was shown illustratively for T1D in **Table 2**.

There is cross-talk between the omega-3 PUFA and the thyroid hormone pathways, as exemplified by the augmented thyroid hormone signaling pathways in the liver, this being one mechanism used by n-3 PUFAs to affect lipid metabolism (108). For instance, specific steps of TH signaling in lipid metabolism that are influenced by n-3 PUFA include higher liver expression of the thyroid hormone nuclear receptor TRβ1 and mitochondrial α-glycerophosphate dehydrogenase (109). Starting from their previous observation showing that plasma free fatty acids concentration in some hypothyroid patients is above the normal range and that this higher concentration is associated with less severe symptoms of hypothyroidism, Makino et al. investigated the effect of highly purified EPA ethyl ester (EPA-E) derived from fish oil on thyroid function in rats with methimazole-induced hypothyroidism (110). They found that oral administration of EPA-E inhibited the reduction of thyroid hormone levels and the change of thyroid follicles in the hypothyroid rats, suggesting that n-3 PUFA may prevent methimazole-induced hypothyroidism. In a Dutch study on 13 patients with hypothyroidism caused by the ablation treatment for well-differentiated TC (111), induction of hypothyroidism decreased PUFA levels in plasma, erythrocytes and polymorphonuclear leukocytes. Another site of interaction between PUFA and thyroid hormones can be the thyroid hormone plasma transport proteins. Several studies have shown that PUFA inhibit thyroid hormone binding to such carrier proteins (112, 113), one practical consequence being increased tissue availability of the biologically active protein-unbound, free thyroid hormone. In addition, studies on the brain of aged rats that were fed fish oil (27% DHA content) for one month showed an approximately 10-fold increase in the expression of transthyretin (114). Transthyretin is the second major thyroid hormone plasma carrier, which is synthesized also in the central nervous system. Since transthyretin also operates as an amyloid-beta protein scavenger, transthyretin overexpression could prevent the formation of amyloid aggregates (114). This study is relevant because decreased PUFA levels, particularly DHA, were detected in elderly subjects and patients with Alzheimer’s disease (AD), and because there is epidemiological evidence for an association between fish consumption and low prevalence of AD (114). Of interest, AD is also characterized by brain inflammation and decreased local concentration of specialized pro-resolving mediators (SPM) (115). SPM are derived from PUFA and are key in the resolution of inflammation. Because of the technical difficulties in investigating the microglia function directly, Wang et al. took advantage of the useful model of peripheral blood mononuclear cells (PBMC) (115). In their randomized, double-blind, and placebo-controlled trial on 204 AD patients, Wang et al. administered a placebo or a supplement of DHA (1.7 g) and EPA (0.6 g) daily for 6 months. At the end of treatment, in those who received the n-3 PUFA, the plasma levels of DHA and EPA levels increased. When the culture medium of PBMC incubated with amyloid-β 1-40 was analyzed, levels of the SPMs lipoxin A 4 and resolvin D1 secreted by PBMCs were decreased in the patients supplemented with placebo, but unchanged in the patients supplemented with n-3 PUFA. Changes in the levels of SPM secreted by PBMC were positively correlated to changes in plasma transthyretin, and to cognitive changes as well (115). In the setting of tissue protection by PUFA, experimental studies in rats by Videla and colleagues showed that the combination of DHA plus thyroid hormone (T3) protects from liver injury through a synergistic action that also involves causing increased intrahepatic levels of resolvins (116–120).

**Table 3** summarizes human studies that link thyroid disorders with the consumption of contaminated fish (17, 18, 121–127). Overall, the consequences are impairment of thyroid function, as measured by serum levels of thyroid hormones and/or TSH, and triggering of thyroid autoimmunity, as measured by serum levels of thyroid autoantibodies. Only studies by Benvenga and colleagues addressed Hg contamination (17, 18). The group of pregnant women who consumed swordfish selectively or predominantly among other fish species ingested the greatest amounts of Hg and had the greatest both serum levels and rates of positivity for thyroid autoantibodies throughout pregnancy compared to other groups of women (17, 18). As a result, the swordfish eaters had the greatest rate of postpartum thyroiditis, since positivity for thyroid autoantibodies is a major risk factor for such autoimmune type of thyroiditis that develops within the first 12 months after parturition (17, 18).

**Table 3:** Studies on the relationship between consumption of contaminated fish and thyroid disorders in humans. *

Reference	Methods and subjects studied	Main findings
Sarkar et al. *2015* (121)	The St. Lawrence River, its estuary, and the Gulf of St. Lawrence are heavily **polluted** with **thyroid disrupting chemicals (TDC)** from **industries**, their effluents, and urbanization in the Great Lakes Watershed and along the river. The west and south coasts are in contact with the Gulf of St. Lawrence (GSL). In studies on blubber samples from harbor porpoises collected in 1989-1991, samples from St. Lawrence were more **contaminated** by older varieties of **persistent organic pollutants (POPs)** than samples from the Avalon Peninsula (tip of east and south coasts of Newfoundland). Another study found **polychlorinated biphenyls (PCBs), dichlorodiphenyltrichloroethane**, and **its metabolites (DDTs)** infrequently consumed **fish (capelin, halibut, tomcod, smelt, herring, flounder), shellfish (shrimp, crab) and mammals (beluga, seal)** caught in the estuary and the GSL. Local **marine products** are a regular diet of the coastal communities of Newfoundland. [In contrast, Newfoundland depends mainly on food imported from the mainland because it has very few agricultural communities, a shorter growing season, and poor soil]. Data on hospitalizations with **hypothyroidism** (from 1998 to 2012) was obtained from the provincial hospital abstracts held at the Newfoundland and Labrador Centre for Health Information (NLCHI).	Mean ± SD **hypothyroidism** rates of the west [91.8 ± 36.7 persons hospitalized with **hypothyroidism** diagnosis per 100,000 population per year] and south coasts [96.3 ± 52.0/100,00/year] were significantly **higher** than in the east coast [51.3 ± 20.2/100,000/year], that is 1.8 and 1.9 times respectively. High levels of **TDC** were detected in marine animals. Hence, consumption of **contaminated seafood** might trigger **hypothyroidism**, the most common cause of which is **autoimmune thyroiditis**. The authors suspected that **marine products** caught from the GSL and **consumed** by communities from the west and south coasts were **contaminated with TDCs**. Such contamination, in turn, could contribute to the development of **hypothyroidism** in these areas.
Schell et al. (122)	The Akwesasne Mohawk Nation has long-lived, fished, planted, and hunted in the St. Lawrence River valley (both USA side and Canada side). Many **industries** had developed along the St. Lawrence River and its tributaries. The Mohawk Nation has relied heavily on **locally caught fish** and game. Some local species of **fish**, birds, amphibians, and mammals have **polychlorinated biphenyls (PCB), p,p ´-DDE, hexachlorobenzene (HCB), and mirex** levels that exceed the tolerance limits for human consumption established by the U.S. Food and Drug Administration. 115 youths (range 10–17 years) were sampled for PCB and their congeners, **TSH, T4, FT4, T3, FT3, TPOAb**. Breast-feeding history was taken into account, with 47 youths having been breastfed.	18 participants (15.6%) had increased **TPOAb** levels (23% of females, 9% of males). The rate of **TPOAb** positivity was similar in the breast-fed group and non-breast-fed group (17.0% vs. 14.7%). Among participants who were breastfed (n=47), those with elevated **TPOAb** levels had significantly higher levels of all **PCB** groupings, except levels of **non-persistent PCBs** which did not differ significantly. Levels of **p,p’-DDE** were also significantly elevated, while **HCB** and **mirex** were similar. Participants who were breastfed had significant, positive relationships between **TPOAb** levels and all **PCB** groupings, except groups comprised of **non-persistent PCBs**, and with **p,p’-DDE, HCB**, and **mirex**. No effects were evident among nonbreastfed young adults.
Turyk et al. (123)	To assess whether **polybrominated diphenyl ethers (PBDE)** body burdens are related to **thyroid** and steroid hormone levels, **thyroid antibodies, and thyroid disease** is frequent and infrequent adult male **sport fish consumers**. A cohort of 4,206 frequent and infrequent consumers of Great Lakes **fish** established during the early 1990s in a previous study, was invited to participate in a follow-up study. 405 adult males were tested for **PBDE congeners, polychlorinated biphenyls (PCB)** congeners, **thyroglobulin antibodies (TgAb), TSH, T3, T4**, and T4-binding globulin (TBG). Data were collected on demographics, **fish consumption,** medical diseases, and medication use.	Data are reported for the 308 men without exclusion criteria. Thus, excluded were also 21/405 men (5.2%) using thyroid hormones or having thyroid disease. **PBDE** were positively related to levels of **T4** and inversely related to levels of **T3** and **TSH**. **PBDE** were positively associated with the percentage of T4 bound to albumin, and inversely associated with the percentage of T4 bound to TBG. Participants with **PBDE** above the 95th percentile were more likely to have **TgAb**, although high **PBDE** exposure was not associated with **thyroid disease**. Indeed, **TgAb** were detected in 7.8% of the full cohort, but in 31.3% of those whose **∑PBDEs** exceeded the 95th percentile [odds ratio (OR) = 6.1].
Bloom et al. (124)	Great Lakes **sportfish anglers** represent a population with potentially elevated dietary exposure to **PBDEs**. 36 licensed anglers who participated in the New York State Angler Cohort Study completed questionnaires regarding demographic, clinical, and **sportfish consumption** information. Archived blood samples were analyzed for **T4, FT4, T3, TSH**, and nine **PBDE** congeners.	There was a positive association between **ΣPBDEs** and **FT4**, which could have been significant with a sample size approximately 9 times greater.
Bloom et al. (125)	Study as above (124), except that the **pollutants** measured in sera of the 36 licensed **anglers** were **polychlorinated dibenzo-p-dioxins (PCDDs), coplanar biphenyls (PCB), dibenzofurans (PCDFs),** and **PCB IUPAC #153**.	There was a significant inverse linear association between the **sum of dioxin-like congener concentrations (∑DIOXs)** and **FT4**.
Hagmar et al. (126)	For the population living in the coastal areas around the Baltic Sea, **consumption** of **locally caught fatty fish** is the main source of **exposure to persistent organohalogens (OHS),** which are **endocrine** disruptors. Persons who consume **great amounts of contaminated fatty fish** from the Baltic Sea may constitute a **risk population**. The aim of this study was to assess whether high dietary **exposure to OHSs** affected hormone levels (among which **FT3, T3, FT4, T4**, and **TSH**) in adult men. Participants were 110 men who **consumed** varying amounts of **fish** (i.e., 0 to 32 meals per month).	In regard to **thyroid function tests**, the only significant association was negative, and consisted of the negative correlation between **2,2’,4,4’-tetrabromodiphenyl ether** and **TSH**.
Langer et al. (127)	In an area of the Michalovce district in East Slovakia, heavy **industrial pollution by polychlorinated biphenyls (PCBs)** developed in 1955-1984 and very high **PCB** levels persist in the environment. Environmental **pollution** occurred because of activities of **chemical factories** manufacturing **polychlorinated biphenyls (PCBs)** and the entirely unlimited **dumping of toxic waste to the nearby Laborec river**. The average **PCBs** level found in the serum of 101 chemical factory employees and persons living nearby reached 7300 ng/g of lipids. In 1998, the average values of organochlorines found in predators (e.g., zander [*Stizostedion lucioperc*a], pike [*Esax lucius*], sheatfish [*Silurus glanis*], perch [*Perca fluviatilis*], and asp [*Aspius aspius*], from the **polluted Sirava lake** and **Laborec river**, were 375430 ng/g lipids for a sum of 15 most abundant **PCB congeners** (upper range limit was 933 770 ng/g which is nearly 1 mg/g lipids) and 15620 ng/g for 2,20-2-bis(4-chlorobiphenyl)-1,1-dichloroethylene (**DDE**). In contrast, the same fish species from the neighboring Ondava river and Domasa lake with relatively background pollution had an average value of only 5150 ng/g for a sum of PCBs and 8420 ng/g for DDE, still considerably higher than the values of PCBs (1100 to 4600 ng/g) reported for the Baltic herring. 2045 adults from the said **polluted area** and the surrounding background **pollution** area were investigated using questionnaire data, **thyroid volume by ultrasound (ThV),** urinary iodine, and serum levels of 15 **PCB congeners, hexachlorobenzene (HCB), α-, β- γ-hexachlorocyclohexane (HCH), 2,2’-bis(4-chlorophenyl)-1,1,1-trichloroethane (DDT), 2,2’-2-bis(4-chlorobiphenyl)-1,1-dichloroethylene (DDE),** and **thyroid indices (TSH, FT4, anti-thyroperoxidase antibodies [TPOAb]).** Information on the frequency of **fish** meals and approximate annual **consumption of fish** from local waters was obtained by questionnaires. Both within the **high pollution area** and the control background area, participants were divided into five groups based on the amount and frequency of **fish consumption**. Comparisons were made between different groups in the same area and between the same group in the two different areas.	In the whole cohort of 2045 participants, those with the **highest fish consumption** had **levels of PCB, DDE**, **and HCB greater** than the corresponding levels of those with **no or low fish consumption**, regardless of the area. However, the same group had greater levels if it belonged to a **highly polluted area**. For instance, in the group with the **highest fish consumption from the polluted area**, **PCB** levels were 4926 ± 971 ng/g lipid compared to 1063 ± 162 in the corresponding group of the background area. Upon continuing the comparison between these two groups, **ThV** was **almost 2 mm greater** (11.62 ± 0.58 vs 10.01 ± 0.05 ml), the **rate of TPOA**b **positivity 3-fold greater** (34% *vs* 10.6%) and the **rate of FT4 levels** above 20 pmol/L **almost 50-fold greater** (14.4% *vs* 0.3%). **TSH** levels were not presented, while they were presented in 16 marital pairs from the **high pollution area** [see below]. In contrast, in such 16 marital pairs, FT4 levels were not presented. The authors concluded for an **association of contaminated fish consumption with very high blood levels of PCBs, HCB and DDE, increased ThV**, **increased frequency of positive TPOAb,** and **high levels of FT4**. These **relationships were confirmed** in 16 marital pairs from the **high pollution area with very high PCB levels** in both members associated with **high fish consumption**. Actually, in these 32 persons, **ThV** was 12.60 ± 1.27 ml, the rate of **TPOAb** was 43.7%, and the rate of **subclinical hypothyroidism** was 10%.
Benvenga S et al. (17)	The Mediterranean Sea (MS) is a semi-closed basin considered to be the most polluted European sea. Top **predators** caught in the MS tend to accumulate high amounts of **toxic metals and other pollutants**. **Swordfish** caught in the main spawning area (Straits of Messina, southern Italy) were contaminated more than **swordfish** caught in the Atlantic Ocean (Azores islands). **Fish consumption** and **serum thyroglobulin antibodies (TgAb) and thyroperoxidase antibodies (TPOAb)** were measured during gestation (first and second trimester) and postpartum (day 4) in 236 thyroid disease-free, nonsmoker Caucasian women with stable dietary habits and stable residence in the Messina province. Women were divided into four groups (A-D) based on the type of **fish consumed**: groups A (n=48; selective or predominant **swordfish consumption**), B (n = 52; selective or predominant **oily fish consumption**), C (n= 68; **swordfish** plus other fish, with **swordfish** consumption accounting for <50% of the total monthly **fish consumption**; if eaten, **oily fish** also accounted for <50% of the total monthly **fish consumption**), D (n = 68; consumption of **fish** other than **swordfish** and **oily fish**).	**Fish consumption** was quantitatively similar in all groups, equivalent to 7.0 ± 2.6 through 7.8 ± 2.1 times monthly. The leading **seafood** in group A (**swordfish**) and group B (**oily fish**) were consumed with the same frequency (6.2 ± 2.2 vs. 6.1 ± 2.5 times monthly). Positivity rates and serum levels of **the two thyroid Ab** were the highest in group A and the lowest in group B. For instance, **TPOAb** positivity at 1st, 2nd trimester of pregnancy and day 4 postpartum was 25%, 17%, and 12% in group A, but always 0% in group B, with intermediate frequencies in groups C and D. Serum levels of **both Ab** were also the highest in group A and the lowest in group B. The **content of mercury** in the **fish consumed** monthly by the four groups was estimated to be the **highest** in group A and the **lowest** in group B (approximately 1000 and 25 μg), with values of approximately 250 and 35 μg in groups C and D, respectively.
Benvenga S et al. (18)	Study as in ref. 17, but on a larger cohort of pregnant women (n= 412) and with a longer follow-up (end of the 12th month postpartum) to permit evaluation of the primary outcome: frequency of **postpartum thyroiditis (PPT)** and its **evolution into permanent hypothyroidism (PH).** Secondary outcomes were serum levels of **thyroid autoantibodies** and, not done in the previous study, **ultrasonography (US) signs of thyroiditis**.	The four **fish** groups remained comparable in terms of frequency of **fish consumption** (7.5-8.0 times monthly or twice weekly). Frequencies of positivity of **TgAb** and **TPOAb**, and serum concentrations of either **thyroid Ab** confirmed those of the previous study (17). **US signs of thyroiditis** during gestation were detected more frequently in group A compared to group B (44.6% and 29.4%), with intermediate values in groups C and D. Overall, the frequency of **PPT** was 15.3%. However, the greatest frequency was recorded in group A (23.9%) and the lowest in group B (4.7%), with intermediate rates in the other groups. Overall, the frequency of **PH** was 54%, with no difference between the four **fish** groups (50 to 56.2%), particularly between **fish** groups A and B (54.5% and 50%).

*Keywords of relevance are highlighted by the bold-face print.

## Fish and Omega-3 PUFA as Protection From Thyroid Disorders

In the preceding section, we have reminded the role of Hg as an autoimmunity trigger (17, 44–51). However, omega-3 PUFA antagonize this effect of Hg (128, 129). Gill et al. (129) showed that dietary ingestion of n-3 PUFA (fish oil) promotes CD95 signaling by upregulating caspase 8 activation and that DHA counteracts the negative effect of Hg on CD95 signaling in T lymphocytes (128).

The clinical benefit of thyroid autoimmune disorders given by consuming Hg-poor, omega-3-rich oily fish or by taking omega-3-based supplements (17, 18, 130–132) is summarized in **Table 4**. The group of pregnant women who consumed oily fish, selectively or predominantly among other species of fish, ingested the lowest amount of Hg but the greatest amount of omega- PUFA; this group of women also had the lowest both serum levels and rates of positivity for thyroid autoantibodies throughout pregnancy compared to other groups of women (17, 18). As a result, the oily fish eaters had the lowest rate of postpartum thyroiditis.

**Table 4:** Studies/reports on the relationship between consuming Hg-poor, omega-3-rich oily fish or by taking omega-3-based supplements and the clinical benefit towards thyroid autoimmune disorders. * ^§^

Reference	Methods and subjects studied	Main findings
Benvenga S et al. (17)	See above, **Table 3**.	**Fish consumption** was quantitatively similar across groups, equivalent to an average of 7 to 8 times a month [see above, **Table 3**]. Positivity rates and serum levels of both **TgAb and TPOAb** were the lowest in group B and the highest in group A. For instance, **TPOAb** positivity at 1st, 2nd trimester of gestation, and day 4 postpartum was always 0% in group B, but 25%, 17%, and 12% in group A, with intermediate frequencies in groups C and D. Serum concentrations of both Ab were also the lowest in group B and the greatest in group B. The **estimated content of mercury** in the **fish consumed** monthly by the four groups was the lowest in group B and the highest in group A (approximately 25 and 1000 μg, respectively), with values of approximately 250 and 35 μg in groups C and D, respectively. In contrast, the **estimated content of omega-3 fatty acids (EPA plus DHA)** in the **fish consumed** monthly was the greatest in group B and the smallest in group A (13.2 ± 5.4 and 6.3 ± 2.1 g, respectively), with values of 6.0 ± 2.8 and 5.1 ± 3.8 g in groups C and D, respectively.
Benvenga S et al. (18)	See above, **Table 3**.	The four **fish** groups remained comparable in terms of frequency of fish consumption (7.5–8.0 times/month or twice a week) [see above, **Table 3**]. Results of frequency of positivity and serum concentration of either **thyroid autoantibody** (**TgAb**, **TPOAb**) confirmed those of the previous study. The lowest and the highest rates of detection of **US signs of thyroiditis** during pregnancy were detected in groups B and A (29.4 and 44.6%, respectively), with intermediate values in groups C and D. Overall, frequency of **postpartum thyroiditis** (**PPT**) was 15.3%. However, the smallest frequency was recorded in group B (4.7%) and the greatest in group B (23.9%), with intermediate rates in the other groups. Overall, the frequency of **evolution of PPT into permanent hypothyroidism (PH)** was 54%, with no difference between the four **fish** groups (50 to 56.2%), particularly between groups B and A (50% and 54.5%).
Breese McCoy, (130)	The author, an adjunct professor of physiology at Oklahoma State University, reports her self-treatment of postpartum **Graves’ disease** with **flaxseed oil**.	“*Approximately one year into the propylthiouracil (PTU) treatment, I became aware that* ***omega-3 fatty acids*** *are thought to reduce the inflammation associated with certain autoimmune disorders, such as rheumatoid arthritis. … With this information in mind, I began a regimen of* ***flaxseed oil supplements****, 5-1,000 mg tablets twice a day.* ***Flaxseed oil*** *is over 50%* ***omega-3 fatty acids*** *(mainly alpha-linolenic acid), but it also contains about 15% omega-6 fatty acids (mainly linoleic acid). Within approximately eight weeks*, ***TSH*** *levels had* ***normalized*** *for the first time (0.31 µIU/mL, reference range 0.30-5.0). PTU was then discontinued, and* ***flaxseed oil*** *tapered to less than half the original dose, but* ***TSH*** *levels slipped below normal again within six months (0.29 uIU/mL, ref. range 0.35-5.00).* ***Symptoms*** *were* ***mild****, and* ***T4*** *was within* ***normal*** *range (1.0 ng/dL, ref. range 0.7-1.9 ng/dL), so I declined to restart PTU therapy*.” … The following year, she was pregnant again. *“… by the fourth week postpartum, my* ***TSH*** *was again suppressed (0.174 µIU/mL, ref. range 0.30-5.00), becoming undetectable by four months postpartum. This time, however, plasma* ***T4*** *remained within* ***norma*** *l range (1.7 ng/dL, ref. range 0.7-1.9 ng/dL). Although the physician advised me to take a low dose of PTU, I was experiencing no noticeable symptoms of hyperthyroidism and declined the prescription due to breastfeeding. As before, I then* ***restarted a flaxseed regimen*** *at about six months postpartum (this time, three tablespoons of whole seed on cereal each morning). My* ***condition began improving****, and plasma* ***TSH normalized*** *within six months (0.57 µIU/mL, ref. range 0.35-5.00).* ***Flaxseed*** *was then discontinued, and there has been no recurrence over the following four plus years*”.
Dolan et al. (131)	Management, with **omega 3** and other nutraceuticals, of a 34-yr-old **Hashimoto’s thyroiditis** woman who had declined thyroid replacement therapy. This patient, who was a part-time worker in a wellness clinic, also had a personal history of seasonal allergies, and a multigenerational **history of thyroiditis and autoimmune disorders**. In addition to supplementation and dietary changes, she performed two acupuncture treatments.	Before being managed by the team, the patient self-prescribed a vegan diet and dietary **supplements**. Such **supplements** consisted of **selenium** (100-200 µg/day), iron, vitamin D3, probiotics, N-acetyl-L-cysteine. At the first visit, the patient reported feeling “*ravenously hungry*” having palpitations, bloating, low libido, low energy, cold hands and feet, and mental sluggishness. The authors switched her to nutritional **supplementation** of vitamins (B complex, D3), coenzyme Q10, α-lipoic acid, zinc, magnesium, **omega-3 oil (DHA/EPA),** L-glutamine, quercetin, and probiotics (50 billion live organisms from 14 strains) in conjunction with a customized herbal tincture [milky oat spikelet (*A sativa*), ashwagandha root (*W somnifera*), holy basil (*O sanctum*), damiana (*T diffusa*), cinnamon bark (*Cinnamomum* spp)] and a customized tea [chamomile flowering tops (*M chamomilla*), ginger (Z officinalis) rhizome, and agrimony (*A eupatorium*) herb]. Noteworthy, the vitamin B complex recommended, and to be taken once daily, contained 40 mg **inositol**. Furthermore, the patient was advised to avoid sensitive foods (gluten and soy) and **increase** her **intake of** berries, **omega-3 rich foods (sardines, wild salmon, walnuts, organic flax),** quality fats (organic: cold-pressed olive oil, coconut oils, butter or ghee), fermented foods (water, cultured coconut milk, kefir), and filtered water. Interpretation is complicated by the multitude of **supplements**, and by the lack of reference ranges for serum **thyroid function tests (TSH, T4, FT4, T3, FT3)** and **autoantibodies (TgAb, TPOAb).** However, serum **TSH declined** from the baseline level of 4. 91 µIU/ml to 1.62 at month 4 and 1.66 μIU/mL at month 12 post-treatment. The corresponding levels of **FT4** were 1.13, 1.1, and 1.02 ng/dL, and those of **FT3** were 3.1, 2.5, and 2.4 [no unit of measure provided, but it should be pg/ml]. Serum **TgAb fell** from 12.0 to 1.4 and 1.1 IU/mL; **TPOAb fell** from 258 to 115 and 24 IU/mL. In parallel with these biochemical changes, **symptoms improved and had disappeared** at the 8-month checkup.
Woźniak et al. (132)^§^	232 **hypothyroid** volunteers (age ≥18 years; median= 27 years; 203 women and 29 men) were asked to provide information on their diagnosis, clinical manifestations of the disease, lifestyles, and use of dietary **supplements** with effect on their health. Supplements were taken by 197/232, with information taken from websites (74%), physicians (52%), family/friends (46%) and social media (43%).	**Hashimoto’s thyroiditis** was diagnosed in 49% of the 232 participants, with 93% taking L-T4. The most popular **supplements** taken were vitamin D (98%), magnesium (21%), **omega-3 acids (15%), selenium** (14%), multivitamins (14%), vitamin B (13%), iron (10%), vitamin C (9%) and zinc (8%). Supplements were taken for a median period of 1.5 years. The most common symptoms experienced included dry skin (64%), cold intolerance (58%), constipation (28%), somnolence (26%), fatigue (23%), hair loss (22%), headache (21%) and mood swings (14%). Patients were stratified into 8 categories based on the **nutraceutical** taken (vitamin D, vitamins B, iron, zinc, multivitamins, **selenium**, **omega-3 acids**), with data summarized as % **of patients reporting a benefit for 8 items** (**decline of serum TSH**, less fatigue, improved memory and concentration, improved hair and nails condition, improved skin condition, improved general well-being, better quality and longer sleep, alignment of menstrual cycles). Overall, 52% of those who took **supplements** reported health **benefits**. In regard to the **omega-3 category**, reported a benefit was improved hair and nails condition (2%), improved general well-being (8%), improved memory and concentration (13%), and a **decline of TSH** (2%). By comparison, selenium users reported a **benefit** for all items (2 to 8%) except the better quality and longer sleep (0%), while vitamin D users, vitamin B users and multivitamins users reported **benefits** for all 8 items (3 to 35%, 2 to 11%, and 4 to 16%, respectively). **A decline of TSH** occurred in each category (from 2% in the **omega-3** to 7% in the vitamin D), except for iron (0%).

*Keywords of relevance are highlighted by the bold-face print.

^§^For internal inconsistencies in the results reported by Woźniak et al, see text.

Concerning Breese McCoy’s study (130), one comment is the limitation given by the fact that only thyroid function tests were monitored, while thyroid autoantibodies were not.

Some comments deserve the questionnaire-based study on 232 hypothyroid patients from Poland (132), a country where 15.8% of women and 2.5% of men suffer from thyroid disease, with an increase of 4.0 percent points in the year 2019 compared to 2014 (132). First, as properly described by the authors, 24% of participants were diagnosed with additional diseases (8% with polycystic ovary syndrome, 4% with depression, and 2% with insulin resistance). Second, there was enormous variability in the 197/232 hypothyroid patients who took supplements. Various was not only the spectrum of supplements taken (Table 4 of their paper) but also the periodicity of taking them (every day, 74%; irregularly, 16%; every other day, 10%). Concerning the main source of information on supplements, patients chose websites (74%), physicians (52%), family and friends (46%), and social media (43%). About two-thirds of participants took supplements according to the leaflet (71%), while one-third according to physicians’ or pharmacists’ guidelines. Results for the 197 participants (85% of 232) who took the supplements are illustrated in Table 1 of their paper (132). In that table, patients were stratified into 8 categories based on the nutraceutical taken (vitamin D, vitamins B, iron, zinc, multivitamins, selenium, omega-3 acids), with data summarized as a percent of patients reporting a benefit for 8 items, the denominator being the said 197 participants. We think that the results of this study (132) should be interpreted cautiously, mainly because it is not possible to distinguish the effect of a given nutraceutical taken alone from that of the same nutraceutical taken in association with others, and because the numbers presented in the said **Table 1** are internally inconsistent. For instance, in the initial text of the Results section, the authors state that zinc supplementation was taken by 8% of the participants, therefore by 16 participants (0.08 x 197). However, upon adding the percentages reported in **Table 1**, the sum is 26, meaning that the number of participants claiming a benefit from zinc supplementation was 51 (0.26 x 197). For the omega-3 users, the sum of percentages is 23, meaning that the number of participants claiming a benefit from such supplementation was 45. Yet, from the initial text in the Results section, omega-3 users had to be 29 or 30 (0.15 x 197 = 29.5). Other obvious limitations of this Polish study (132) are the lack of details on the degree of hypothyroidism (overt, subclinical, either), the magnitude of change in serum levels of TSH, and having neglected to obtain information on changes in serum thyroid autoantibodies.

Concerning the mechanism behind the protection from/amelioration of AIT and the decrease in serum levels of thyroid autoantibodies caused by the omega-3 PUFA, two recent studies point to the involvement of resolvins (133, 134). Resolvins, lipoxins, maresins, and protectins are specialized pro-resolving mediators (SPM), which are downstream derivatives of PUFA (133). Resolvins of the E series (RVE1-RVE3) derive from EPA, while resolvins of the D series (RVD1-RVD6) derive from DHA. Concerning RVE-1 (5S,12R,18R-trihydroxy-eicosapentaenoic acid), which is the resolvin investigated by Song et al. (133), it triggers all aspects of the pro-resolution cascade, from inhibiting lymphocyte aggregation at the inflammation site to efferocytosis (that is, the process by which apoptotic cells are removed by phagocytic cells). The cell receptor for REV-1 is chemokine-like receptor 1 (chemR23 or CMKLR) (135), with the ligand-receptor interaction triggering anti-inflammatory responses that include marked inhibition of proinflammatory cytokines and chemokines (136). In one article, Song et al. (133) measured serum levels of REV-1 and serum thyroid parameters (TSH, T4, FT4, FT3, TPOAb, and TgAb) in 30 untreated HT patients (median and interquartile range [IQR] of TSH= 2.62, 1.95–3.05 µIU/ml) and 27 age- and sex-matched healthy controls (TSH= 2.38, IQR= 1.54–2.94, P= 0.178 vs. patients). Levels of RVE1 in patients were significantly lower compared to controls (median and IQR= 24.09, 15.76–34.38 pg/mL vs 28.51, 20.76–51.23 pg/mL). RVE1 levels correlated inversely with increasing TgAb levels in both the unadjusted model (OR= 0.945, P= 0.002) and adjusted models (OR= 0.938, P= 0.005). RVE1 levels were the lowest (19.21, 13.81–27.34 pg/mL) in the highest quartile group of TgAb levels (TgAb >361 IU/mL), and the highest (37.70, 24.66–99.16 pg/mL) in the lowest quartile group (TgAb <34 IU/mL) (19.21, 13.81–27.34 pg/mL vs 37.70, 24.66–99.16 pg/mL). Concerning TPOAb, the association with serum levels of REV1 was an inverted U-shaped curve with the lowest RVE1 levels (median and IQR= 19.21, 15.11–26.01) in the highest quartile of TPOAb levels (>431 U/mL) and the highest 31.39 (23.79–62.31) in the second quartile of TPOAb levels (13.6–106 U/mL). RVE1 levels correlated negatively also with T3 and FT3 (133). In another article, Song et al. (134) addressed RVD1 (7S, 8R, 17S-trihydroxy-4Z, 9E, 11E, 13Z, 15E, 19Z-docosahexaenoic acid) by measuring its serum levels in 30 patients with HT and 33 healthy controls. Serum concentrations of RVD1 in patients were significantly lower than in controls (median and IQR= 134.76, 85.35-201.36 pg/mL vs 187.64, 131.01-326.85 pg/mL). There was a significantly negative correlation between the TPOAb levels and the RVD1 levels. The authors also measured serum levels of the inflammatory chemokine IP-10/CXCL-10, and found that this chemokine had a significant negative association with TPOAb in the patients (134). Noteworthy, the administration of DHA (daily doses of 300 mg/kg) combined with T3 (0.05 mg/kg) to rats increased significantly the content of RVD1 and RVD2 in the liver, but left unchanged that of RVE1 and RVE2, indicating a synergistic effect compared to treatment with DHA alone and T3 alone (118). The authors concluded that co-administration of T3 and DHA enhances the capacity of liver for the resolution of inflammation by increasing RVD1 and RVD2 availability, so such co-administration constitutes an important hepatoprotective protocol for clinical purposes (118).

## Omega-3 PUFA-Based Nutraceuticals in the Setting of Thyroid Disorders: Experimental Setting

Experimental studies that have evaluated the thyroidal effects of the omega-3 PUFA (137–143) are summarized in **Table 5**. Omega-3 PUFA were shown to have a neuroprotective (138, 139) and cardioprotective (143) action on unfavorable effects caused by thyroid hormone deficiency. On the other hand, the omega-3 PUFA mitigate, ameliorate certain hyperthyroidism-induced unfavorable effects, such as arrhythmias (141) and hepatic dysfunction (137). Such favorable effects of the omega-3 PUFA on consequences in the peripheral tissues that are caused by opposite states of thyroid dysfunction confer to the omega-3 PUFA a modulatory role that is reminiscent of another dietary nutrient, L-carnitine. Indeed, beneficial effects of L-carnitine were reported both in the hyperthyroidism setting (144, 145) and in the hypothyroidism setting (146, 147), with a modulatory role also demonstrated in the tissue glucocorticoid hormone action (148).

**Table 5:** Studies in experimental animals on the effects of the omega-3 polyunsaturated fatty acids (PUFA) in the thyroid setting. *

Reference	Aims and animals studied	Main findings
Soukup (137)	To review whether i) administration of **n-3 PUFA** could improve thyroid hormone (**TH**)**-induced** pathophysiological changes such as cardiac tissue remodeling and cell-to-cell communication changes, skeletal muscle protein alterations, alterations in the expression of protein kinases, oxidative stress markers, and cell death, mitochondrial functions, changes in serum lipid levels, in activities of key enzymes of **TH** metabolism and acetylcholine esterase or membrane anisotropy, as well as in thermal sensitivity and mobile behavior.	Soukup et al. think that there is a rationale for **n-3 PUFA** administration being capable to **ameliorate TH-induced** pathophysiological changes in settings such as cardiac disorders. For instance, **n-3 PUFA intake** significantly **reduced cardiovascular risk factors**, as they suppressed the incidence of ventricular fibrillation and facilitated sinus rhythm restoration in spontaneously hypertensive **rats** (SHR) in the early and late stages of hypertension. The **antiarrhythmic effects of n-3 PUFA** can be attributed to the attenuation of abnormal myocardial gap junction protein connexin 43 (Cx43) distribution, expression, and phosphorylation, as well as to positive modulation of PKCϵ and PKCδ signaling and normalization of myosin heavy chain (MyHC) profiles. Indeed, intercellular Cx43 gap junction channels are involved in the increased susceptibility of the heart to arrhythmias caused by increased **TH levels**; the expression of PKCϵ, which directly phosphorylates Cx43, is affected.
Sinha et al. (138)	A **murine model of hypothyroidism**-induced neuronal apoptosis was used to investigate the role of **omega-3-fatty acids (ω-3 FAs)** in regulating neuronal apoptosis during brain development. Pregnant and lactating **rats** with methimazole-**induced primary hypothyroidism** were supplemented with a mixture of **EPA** and **DHA**. Apoptosis was studied on cerebella from postnatal day 16 (d16) pups.	In the **hypothyroid** pups, **Ω-3 FA-supplementation** did not significant. The percentages of **DHA** and **EPA** in total cerebellar **FAs increased** significantly in the **ω-3 FA-treated hypothyroid** pups compared to euthyroid pups (Ep) and **untreated hypothyroid** pups (**UHp**). The cerebellar weight of **ω-3 FA-treated hypothyroid** pups was similar to that of Ep and greater than that of **UHp**. In the developing cerebellum of the **hypothyroid** pups, **Ω-3 FA-supplementation decreased** significantly DNA fragmentation and caspase-3. The **protection** given by **ω-3 FAs** was associated with their ability to **prevent** increased levels of pro-apoptotic basal cell lymphoma protein-2 (Bcl-2)-associated X protein (Bax) in the cerebellum during **hypothyroidism**. **Ω-3 FAs increased** the levels of anti-apoptotic proteins like Bcl-2 and Bcl-extra-large (Bcl-x(L)), which were low in **UHp**. **Ω-3 FAs** also **restored** levels of cerebellar phospho (p)-AKT, phospho-c-Jun N-terminal kinase (p-JNK), and phospho-extracellular regulated kinase (p-ERK), which were low in **UHp**. In sum, data support the anti-apoptotic role of **ω-3 FAs** during cerebellar development.
Pal et al. (139)	To study the effects of **iodine** and **n-3 fatty acids [FA]),** separately and together, in the progeny of an **iodine-deficient (ID)** pregnant **rat** model. The supplementation diets were: (i) low-**iodine** diet (LID), (ii) LID+**potassium iodide (KI),** (iii) LID+**n-3 FA**, and (iv) LID+**KI**+**n-3 FA**. Morphological and biochemical parameters at the peak of cerebellar histogenesis on postnatal day 16 (P16) and for both neurobehavioural and motor coordination parameters at P40 were studied.	**n-3 FA** significantly **improved** morphological, functional and biochemical indices of the developing cerebellum, despite no improvement in circulating **thyroid hormone levels**. **Co-supplementation with n-3 FA and iodine rescued** the loss of neurotrophic support, and **salvaged** motor coordination, learning and memory. This additive effect resulted in significantly **improving** neurotrophic support and seemed to be mediated by parallel significant **increase in TH receptor (TR)α** and **normalization of TRβ**, p75 neurotrophin receptor and retinoic orphan receptor α, as well as **prevention** of apoptosis and **strengthening** of anti-oxidative defense. Thus, **n-3 FA** may play an important **mitigating role in iodine deficiency** in **enhancing TH** nuclear receptor-mediated signaling in the developing cerebellum.
Abd Allah et al. (140)	To study the effect of **hypothyroidism** on memory and spatial learning in adult male **rats** (n= 30), the underlying mechanisms and the possible therapeutic value of **omega-3 supplementation**. Rats were divided into three groups; **hypothyroid**, **omega-3 treated** and controls.	**Omega-3 supplementation increased** serum total antioxidant capacity, **decreased** the structural changes of the hippocampus (diffuse vacuolar degeneration and distortion of the pyramidal cells), and **decreased** the expression of Cav1.2 (the voltage-dependent LTCC alpha 1c subunit) protein, and improved memory deficits. Thus, **omega-3** could be useful **neuroprotective** agents against the cognitive impairment caused by **hypothyroidism**.
Gomaa et al. (141)	To investigate, in adult male **rats,** (i) the **hyperthyroidism-induced** hepatic dysfunction (ii) whether such dysfunction could be ameliorated by the **administration of omega-3 on hyperthyroidism-induced** hepatic dysfunction, and (iii) the underlying mechanisms of this ameliorative effect. Rats (n= 24) were divided into three groups; control (which received water for 6 weeks), **hyperthyroid** (which received **L-T4** orally for 6 weeks) and **hyperthyroid omega-3 treated** (which received L-T4 for 2 weeks and then **co-treated with L-T4** and an **omega-3** oral **mixture** of **EPA**+**DHA** for 4 weeks).	The **hyperthyroid omega-3 treated** group had significantly **increased** final body weight and body weight gain, **decreased** liver weight to body weight ratio, **decreased** serum T3 level, **increased** serum TSH level, **decreased** serum levels of transaminases, and TNFα, decreased hepatic levels of total peroxide and IL-1β and increased hepatic levels of total antioxidant capacity when compared with the **hyperthyroid** group. Liver histopathology also confirmed marked **improvement** of the lesions caused by **hyperthyroidism**. In brief, **omega-3** has encouraging **therapeutic effects** against **hyperthyroidism-induced** hepatic dysfunction. These effects can be attributed to multiple mechanisms: **anti-inflammatory, antioxidant, and anti-fibrotic effects**.
Rauchová et al. (142)	To investigate whether a 6-week **supplementation** with **n-3 PUFA** (200 mg/kg of body weight/day intragastrically) would affect lipid metabolism in Lewis male **rats** with **altered thyroid status**.	**Supplementation of n-3 PUFA** did not significantly modify plasma lipid levels in **any thyroid status**. Also, **n-3 PUFA** did not modify **thyroid dysfunction-induced** altered plasma glucose levels.
Awumey et al. (143)	To evaluate some heart parameters in **hypothyroid rats** and euthyroid controls that had received diets enriched in either **n-6** or **n-3 fatty acids (FA).**	In **hypothyroid** animals fed the **n-3 diet,** maximum tension was 105% greater than resting compared to 399% in controls. Similar responses to noradrenaline and adrenaline were observed, that is, maximum tension was significantly greater in both **hypothyroid** and euthyroid rats fed the **n-3 diet**, but the tension was depressed in the **hypothyroid** rats. Binding of the β-adrenoceptor antagonist [3H]-dihydroalprenolol to ventricular membranes had high affinity and was saturable, regardless of **thyroid status** and **diet**. However, binding affinity (Kd) was higher in **hypothyroid** rats fed the **n-6 diet**. The inotropic response to forskolin was the same in **hypothyroid** animals, regardless of **diet**, but the maximum developed tension was significantly greater in euthyroid rats fed the **n-6** compared to the n-3 diet. The dose-response curve for forskolin was shifted to the right in **hypothyroid** rats fed the **n-3 diet**, indicating decreased sensitivity. In sum, the depressed contractility of the **hypothyroid** heart may be attributed in part to an altered lipid environment of the β-adrenoceptor complex. Moreover, **n-3 FA supplementation** can significantly **increase** maximum developed tension in the **hypothyroid** state.

*Keywords of relevance are highlighted by the bold-face print.

## Protection From Thyroid Disorders Given by Nutraceuticals Other Than the Omega-3 PUFA

The very few studies available in the English-language literature on the omega-3 PUFA-based nutraceuticals in the clinical setting of autoimmune thyroid disorders (130, 131) (**Table 4**) contrast with the large number of studies that have tested seleno-L-methionine alone (149–151) or seleno-L-methionine combined with myo-inositol (151–157), which is a hexahydroxycyclohexane (C_6_H_12_O_6_) and one of the nine stereoisomers of inositol that plays a pivotal role in many metabolic pathways (158). In the studies with seleno-L-methionine combined with myo-inositol, the benefit was generally greater than that given by seleno-L-methionine alone or myo-inositol alone. Such benefit was based on the reduction of TSH, TgAb, TPOAb levels, and, when studied (154), on the reduction of serum chemokine levels (CXCL10/IP-10). Myo-inositol+seleno-L-methionine treatment protected blood mononuclear cells (PBMC) of either HT or healthy patients from H2O2–induced stress. Furthermore, the association of myo-inositol and seleno-L-methionine decreased the expression of the chemokines CXCL10/IP-10, CCL2/MCP-1 and CXCL9 (159). In another study, which evaluated the thyroid toxicity of cadmium and protection from this toxicity by nutraceuticals (160), Cd induced a marked overexpression MCP-1/CCL2 and CXCL10 in the thyroid. Again, the protection given by seleno-L-methionine combined with myo-inositol was significantly greater than that of either nutraceutical alone (160). *In vitro* experiments on blood cells or other cells have shown that omega-3 PUFA decrease the secretion of CXCL10/IP-10 (161–163), CXCL9 (164) and CCL2/MCP-1 (11, 165–176).

Another nutraceutical, *Aloe vera* (also known as *Aloe barbadensis*), decreased serum levels of thyroid antibodies and improved subclinical hypothyroidism (177). It is pertinent to remind *Aloe vera* because, among the several nutritional substances contained in its leaves, there are selenium and PUFA, including the omega-6 linoleic acid and the omega-3 linolenic acid, the latter being the second most abundant after one saturated fatty acid (palmitic acid) (178). Noteworthy, in the *Aloe vera* juice used in the study by Metro et al. (177) the fat content is 0.2%, with saturated fatty acids being absent. As written in that paper (177), “*one of the authors decided to take Aloe Barbadensis Miller juice (ABMJ), at the dose of 50 ml every morning on an empty stomach, as a skin soother and laxative*”. This author had a history of HT-associated subclinical hypothyroidism, which she monitored with periodical checks of serum thyroid function tests and thyroid autoantibodies. “*At the biochemical check performed three months after having started taking ABMJ, she was struck by the remarkable improvement of all indices *(**Table 1**)*. The improvement was even more impressive six months later*” (177). Based on this experience, the effects of ABMJ administration were tested, in a 9-month duration trial, on HT women with levothyroxine-untreated subclinical hypothyroidism and high levels of TPOAb (n= 30). Controls were 15 HT women with untreated subclinical hypothyroidism who were matched for age and baseline levels of TPOAb, TSH, FT4 and FT3. In the *Aloe*-treated group, TSH, FT4 and TPOAb improved significantly already at month 3 and further (−61%, +23% and −56%, respectively) at month 9. At baseline, 100% of women had TSH > 4.0 mU/L and TPOAb > 400 U/ml, but at month 9 rates fell to 0% and 37%, respectively. In contrast, the control group had no significant changes in any index (177).

Of the three aforementioned chemokines (CXCL10/IP-10, CCL2/MCP-1 and CXCL9), only one (MCP-1) was evaluated in terms of response to *Aloe vera*, and only by two studies (179, 180). In one study (179), a wound dressing containing an *Aloe vera* extract plus gelatin decreased the production of MCP-1 by 75% in human mesenchymal stem cells treated with TGFβ. In the other study (180) the oral administration of two antidiabetic phytosterols isolated from *Aloe vera* lophenol (lophenol and cycloartanol) decreased serum and hepatic concentrations of MCP-1 in Zucker diabetic fatty (ZDF) rats. Nevertheless, there is abundant literature on *Aloe vera* being able to decrease the production of pro-inflammatory cytokines and chemokines (181–188).

## Omega-3 PUFA and Oily Fish in the Nutraceutical Market

Based on an article of May 2018 by the main Italian press agency ANSA (189), Italy is the first European country for nutraceutical products based on per capita expenditure, that is, euro 40 compared to euro 28 of the EU. Italians spend more than euro 3.2 billion on dietary/supplements/nutraceutical products. A major driver for this expenditure is wishing to prevent and/or treat metabolic diseases (189) Based on the survey “*The Italian food supplement supply chain, 2019-2020*” by the FederSalus Research Centre (that is the national agency of the Italian producers and distributors of food supplements), in late 2019, the food supplement market in Italy reached a value of approximately euro 3.6 billion (190), corresponding to 27% of the euro 13.2 billion value of the European market, thus preceding Germany and France (18 and 8%, respectively). In particular, 32 million persons used supplements (65% of the adult Italian population) and 261 million packs (equal to 8 per capita) were sold in 2019 (190). At Italian pharmacies, the main sales channel, supplements are confirmed to be the second category after prescription drugs and give the greatest boost to growth, with 28.6 million medical prescriptions issued for food supplements in 2019 (190). Interestingly, the leading medical category that accounts for most of the 28.6 million medical prescriptions is represented by the general practitioners (21%) (190). Endocrinologists are absent in the first 8 positions, but the document fails to specify which categories are represented in the 9th category (“others”), a category that accounts for 12% of the prescriptions (190). In terms of comparison with the United States, based on data from a decade ago, annual supplement sales were USD 23 billion, with approximately 40,000 supplements products being on the market (191). In 2015, the American market for supplements was valued at USD 37 billion (151), but expected to reach USD 56.7 billion by 2024 (192) or USD 117.92 billion by 2027 (193).

With regard to the omega-3 products, their global market was valued at USD 2.10 billion in 2020 (USD 554.8 million in the US alone) (194), and it is projected to go up at a compound annual growth rate (CAGR) of 7.4% during the from 2020 to 2025 (195) and 7.8% from 2020 to 2028 (194). The main factors responsible for such increased consumption include the rising frequency of cardiovascular diseases (CVDs), changing dietary habits, the rising importance of immunity development post-COVID-19 and an increasing number of omega-3-based pharmaceutical product launches. DHA dominated the market in 2020, but EPA is expected to grow faster because of the increasing demand for immunity-boosting supplements (194).

The marine source segment of omega-3 supplements held the largest revenue share of over 82.8% in 2020. Fish oil (which contains both DHA and EPA) is the major marine source, and it is derived mainly from anchovy fish. Prices of omega-3 PUFA reflect the extraction and processing costs of fish oil, and they change depending on the availability of anchovy fish. Increasing contamination of fish by Hg and other pollutants is expected to negatively affect the prices of fish oil (194). Fish oils are currently considered the best and generally a safe source of omega-3 (196). However, the declining fish population due to overfishing has led to searching for more sustainable sources. Krill oil (which contains both DHA and EPA) and algae oil (which contains only DHA) have gained greater importance in recent years as alternatives, with algae serving as an option for the vegetarian population (197). In the US market, 9% of grocery shoppers buy high-omega-3 food or beverages in a typical shopping trip, with the proportion of adults who take fish oil supplements have increased from 8% in 2006 to 17% in 2011 (198). Omega-3 PUFA are also derived from plant sources including walnuts, pumpkin seeds, soybean oil, flaxseed oil, and canola oil, with plant oils being major sources of alpha-linolenic (ALA).

By comparison, the European market of the omega-3 products during the period (2019–2024) is expected to reach USD 14.61 billion by 2024 growing at a CAGR of 7% (199). The European demand for DHA is forecasted to increase significantly due to the favorable regulations in the European Union, which made DHA a mandatory ingredient in infant formula from 2020. Chia seeds are gaining popularity in Europe because of their nutritional and health properties that derive from their content in omega-3. Germany is the top European importer of chia seeds, preceding the Netherlands, Spain, and the United Kingdom (199).

In 2010, Friend of the Sea (FoS), an international nongovernment organization with the mission of promoting environmental conservation, introduced specific standards for producers of fish oil, fishmeal, fish feed and omega-3 supplements (200). Accredited third-party certification bodies certify that the oil originates from fisheries that are compliant with FoS sustainable fishing requirements, and that a full chain of custody occurs throughout the supply and the production chain (200). As of September 2018, 439 companies (compared to only 76 in 2015) adhere voluntarily to FoS standards for fish oil, fishmeal, fish feed and omega-3 supplement. Certified oils originate mostly from approved Peruvian anchovy fisheries and fleet (*Engraulis ringens*, 29%), Antarctic krill (*Euphausia superba*, 22%), European sardine (*Sardina pilchardus*, 8%), European anchovy (*Engraulis encrasicolus*, 7%), Chub mackerel (*Scomber japonicus*, 7%), Atlantic cod (*Gadus morhua*, 3%) (195). FoS presence in the nutraceutical industry has grown considerably in the United States, where it now accounts for over 50% of total FoS certified supplements (268 companies) (195). The United States are followed by France, Canada, Norway, United Kingdom and Italy, these six countries representing the top 6 countries for FoS labeled supplements.

Some studies are described now to illustrate the bioavalability of different types of omega-3 products. A 4-week randomized, placebo-controlled, double-blinded Icelandic study on 99 adults (of whom 77 completed the study) investigated the bioavailability of LC n-3 PUFAs from microencapsulated powder compared with ready-to-eat meals enriched with liquid fish oil (201). Participants were randomized into three groups 38 received 1.5 g/d EPA and DHA as meals enriched with liquid fish oil; 30 received the same amount of these LC n-3 PUFA as microencapsulated fish oil powder and regular meals; and 31 (controls) received placebo powder and regular meals. The authors found similar bioavailability between ready-to-eat meals enriched with liquid fish oil and LC n-3 PUFAs in encapsulated powder (201).

Starting from the fact that no conclusive information had been published on the relative bioavailability of omega-3 supplements taken as fish oil or krill oil, with few studies suggesting that the phospholipid form (krill) is absorbed better than the fish oil ethyl ester (EE) or triglyceride (TG) forms, Yurko-Mauro et al. (202) compared the oral bioavailability of the same dose of both DHA and EPA in fish oil-EE vs. fish oil-TG vs. krill oil after a four-week supplementation. In this double-blind, randomized, parallel study, 66 healthy adults were supplemented with a 1.3 g/day dose of EPA+DHA (approximately 816 mg/d EPA + 522 mg/d DHA, regardless of formulation) as either fish oil-EE (n=22 participants), fish oil-TG (n=22) or krill oil capsules (n=22). The authors found similar plasma and red blood cell levels of EPA+DHA across fish oil and krill oil products when matched for dose, DHA and EPA concentrations, indicating comparable oral bioavailability irrespective of formulation (197). For instance, mean total plasma DHA+EPA at 672 h were 90.9 ± 41 µg/mL (fish oil-EE), 108 ± 40 µg/mL fish oil-TG (fish oil-TG), and 118.5 ± 48 krill oil (krill oil), with a probability value just borderline significant (P= 0.052). Indeed, upon perusing figures in this paper (202), a decreasing hierarchy is evident for both plasma and red blood cell levels of DHA+EPA across all time points: krill oil > fish oil-TG > fish oil-EE. Furthermore, in a Canadian double-blinded, randomized, placebo-controlled, crossover trial on 24 volunteers, krill oil resulted more effective than fish oil in increasing n-3 PUFA both in plasma (P = 0.0043) and in red blood cells (P = 0.0011) (203). This study consisted of three treatment phases including krill or fish oil, each providing 600 mg of n-3 PUFA or placebo control, corn oil in capsule form (203).

DHA and EPA are added to several commercially available foods, such as infant and pet formulas, and they are also supplemented in animal feed to incorporate them in consumer dairy, meat, and poultry products (204). The main sources of EPA and DHA are fish oils or purified preparations from microalgae (204). In a one-month duration Australian study (205), 16 healthy males were provided with a range of foodstuffs naturally containing LC n-3 PUFA (fresh and canned fish, canola oil, flaxseed meal) and items fortified with fish oil (sausages, milk, margarine spread, luncheon meat, French onion dip); food choices were left to the discretion of each participant. Blood and cell levels of ALA, EPA and DHA increased highly significantly after 4 weeks (P<0.001) (205).

## Conclusions

Because of the systemic action of thyroid hormones and their pleiotropic effects, thyroid disrupting chemicals (such as those mentioned in **Table 3**) represent a public health issue, making the comprehension of the mechanisms through which they interfere on thyroid homeostasis considerably importance.

Considering (i) the increasing incidence of both HT and TC worldwide (206–213); (ii) the aforementioned predisposing role for TC (particularly, papillary TC) and its advanced stages exerted not only by HT but also by even trendwise high serum TSH levels *per se* (65–88); (iii) the risk for developing metabolic and cardiovascular disorders conferred by both elevated/trendwise elevated serum TSH levels and thyroid autoimmunity (214–230), then it would be beneficial to contrast the appearance and/or duration of HT as well as to correct the slightly elevated serum TSH levels of subclinical hypothyroidism, the leading etiology of which is AIT. Furthermore, HT is frequently associated with other endocrine and nonendocrine autoimmune diseases, on which omega-3 PUFA proved to be beneficial [see above, Introduction]. For instance, 19.5% of 3,069 HT patients had evidence of at least one other autoimmune disease compared to 3.6% of 1,023 patients with multinodular goiter (231). As a corollary, there would be the place for the use of nutraceuticals to prevent/delay/minimize the onset/burden of autoimmune thyroiditis and the magnitude of TSH elevation. Taking into account their aforementioned anti-autoimmunity and anti-cancer (including TC) properties, the omega-3 PUFA would be appropriate nutraceuticals to be used, either alone or combined with other supplements.

## Author Contributions

Writing - original draft: SB and FF. Writing - review and editing: SB, FF, LP, AA, GB, FV and MM. Supervision: SB, FV and MM. All authors have read and approved the final manuscript and all materials before submission.

## Conflict of Interest

The authors declare that the research was conducted in the absence of any commercial or financial relationships that could be construed as a potential conflict of interest.

The reviewer SF declared a past collaboration with the authors AA, SB to the handling editor.

## Publisher’s Note

All claims expressed in this article are solely those of the authors and do not necessarily represent those of their affiliated organizations, or those of the publisher, the editors and the reviewers. Any product that may be evaluated in this article, or claim that may be made by its manufacturer, is not guaranteed or endorsed by the publisher.
